# Urinary Bladder Cancer Susceptibility Markers. What Do We Know about Functional Mechanisms?

**DOI:** 10.3390/ijms140612346

**Published:** 2013-06-10

**Authors:** Aleksandra M. Dudek, Anne J. Grotenhuis, Sita H. Vermeulen, Lambertus A. L. M. Kiemeney, Gerald W. Verhaegh

**Affiliations:** 1 Department of Urology, Radboud University Medical Centre, Geert Grooteplein 16, Nijmegen 6525 GA, The Netherlands; E-Mails: b.kiemeney@ebh.umcn.nl (L.A.L.M.K.); g.verhaegh@uro.umcn.nl (G.W.V.); 2 Department for Health Evidence, Radboud University Medical Centre, Geert Grooteplein 21, Nijmegen 6525 EZ, The Netherlands; E-Mails: a.grotenhuis@ebh.umcn.nl (A.J.G.); s.vermeulen@ebh.umcn.nl (S.H.V.); 3 Nijmegen Centre for Molecular Life Sciences, Geert Grooteplein 28, Nijmegen 6525 GA, The Netherlands; 4 Nijmegen Centre for Evidence Based Practice, Geert Grooteplein 21, Nijmegen 6525 GA, The Netherlands

**Keywords:** bladder cancer, GWAS, risk variants

## Abstract

Genome-wide association studies (GWAS) have been successful in the identification of the several urinary bladder cancer (UBC) susceptibility loci, pointing towards novel genes involved in tumor development. Despite that, functional characterization of the identified variants remains challenging, as they mostly map to poorly understood, non-coding regions. Recently, two of the UBC risk variants (*PSCA* and *UGT1A*) were confirmed to have functional consequences. They were shown to modify bladder cancer risk by influencing gene expression in an allele-specific manner. Although the role of the other UBC risk variants is unknown, it can be hypothesized—based on studies from different cancer types—that they influence cancer susceptibility by alterations in regulatory networks. The insight into UBC heritability gained through GWAS and further functional studies can impact on cancer prevention and screening, as well as on the development of new biomarkers and future personalized therapies.

## 1. Introduction

For many years, the identification of disease-susceptibility genes was based on linkage studies in high-risk families. Although rare, high-penetrance mutations have been identified, most of the genetic susceptibility in sporadic cases remained unexplained. Recently, however, genome-wide association studies (GWAS) have been performed for many complex diseases, including cancer. Cancer is a multifactorial disease, arising from the complex interplay between genetic and environmental factors. The GWAS approach allows a search for novel susceptibility loci throughout the genome in a hypothesis-free manner [1]. Most of the identified variants are common and confer small risk (odds ratios between 1.1 and 1.3). However, the involvement of many low-penetrance loci can explain differences in cancer susceptibility between individuals [2]. Despite the large number of identified risk variants, only a modest number of functional studies have so far been published. The majority of identified genetic variants map to non-coding regions in the genome and require fine-mapping, and therefore finding the causative variants, as well as understanding their functionality and role in cancer development, remains challenging [3].

Urinary bladder cancer (UBC) is a common malignancy of the urinary tract, with 180,500 estimated new cases each year and 38,200 deaths in the European Union [4], and with 73,510 estimated new cases and 14,880 deaths in United States [5]. Despite many years of research and the identification of several genes involved in bladder cancer pathogenesis, the large part of its heritability remains unknown [6]. In the past, many hypothesis-driven candidate gene studies were performed. However the majority of results were not reproduced, with the exception of *GSTM1* and *NAT2* [1]. GWAS has successfully identified several bladder cancer risk variants. Understanding the functional consequences of these single nucleotide polymorphisms (SNPs) will help in understanding the role of the identified variants in bladder carcinogenesis. This can lead to the development of new biomarkers or therapies for selected patients, making personalized medicine for bladder cancer reality [7].

The aim of this review is to give a comprehensive overview of the bladder cancer risk of SNPs and the functional consequences of these genetic variants. We will include some functional studies performed for the risk SNPs that are located in UBC susceptibility regions, but were performed in relation to other cancer types. Finally, we will suggest some future research lines that will improve our understanding of the heritability underlying UBC.

## 2. Bladder Cancer

Urinary bladder cancer occurs frequently, with the highest incidence rates in developed countries. It is typically diagnosed in older patients (55 years of age or older) [8]. Up to 95% of the patients develop urothelial cell carcinoma. The remaining types include adenocarcinoma, squamous cell carcinoma and other rare histological types [9]. Urothelial cell carcinoma also can include such histological variants, which can influence prognosis [10]. About 75% of patients are diagnosed with non-muscle-invasive bladder cancer (NMIBC), containing the clinical stages Ta, T1 and CIS (carcinoma *in situ*). The Ta stage is characterized by the presence of noninvasive papillary lesions of low and high grade, of which the latter tends to recur most frequently. T1 tumors invade into the subepithelial connective tissue but not into the muscle layer. T1 tumors harbor a higher risk of progression to muscle-invasive disease, and even come into consideration for cystectomy. CIS is a flat high-grade lesion and has a higher progression rate to muscle invasive bladder cancer (MIBC) [11]. The treatment of NMIBC involves the removal of the tumor by transurethral resection (TUR). Surgical removal is usually followed by one, or a course of intravesical installations of chemotherapy or immunotherapy, which reduce the risk of recurrence. However, the impact on progression and cancer-specific survival remains uncertain [12].

The remaining 25% of patients are diagnosed with MIBC (T2 stage or higher). Due to the high rate of progression to metastatic disease, MIBC, if still confined to the bladder, is mostly treated by radical cystectomy [13], but systemic chemotherapy, and radical radiotherapy are also widely used in some countries [14].

The main risk factor for developing bladder cancer is tobacco smoking, accounting for an estimated 50% of cases among men and 35% of cases among women [15]. Smoking cessation reduces the risk of developing bladder cancer immediately. However, due to the fact that many carcinogens are present in tobacco smoke, which alter gene expression and damage the DNA, the increased risk is still present even after 25 years [16]. Bladder cancer risk is also correlated with various occupational (mainly aromatic amines) and environmental exposures (e.g., arsene in drinking water). Other risk factors include exposure to ionizing radiation, chronic inflammation or schistosomiasis [17].

## 3. Genome-Wide Association Studies

The environmental risk factors for developing bladder cancer, such as smoking, are common, although only a fraction of people exposed to them will eventually suffer from this disease. This suggests the contribution of genetic variation in determining bladder cancer risk [18]. In recent years, GWAS have emerged as a powerful approach in the discovery of genetics underlying complex traits like cancer.

### 3.1. Principle of GWAS

Until now, over 1500 genome-wide association studies have been performed [19], allowing to test, in an unbiased way, the most common genetic variation (SNPs) for the association with particular, complex traits. GWAS is usually based on the case-control design. Both controls and cases affected by the particular trait of interest are genotyped for a large set of SNPs (tagSNPs), spaced across the genome, using commercial chips. These proxy markers represent the regions of strong linkage disequilibrium (LD), characterized by a high correlation (*r*^2^ > 0.8) between SNPs. Therefore, genotyping of particular tagSNPs can give information about other SNPs in high LD with the measured one. Because of the large number of analyzed loci there will be many false positive findings in the discovery set. Particular standards have been adopted in the field. For example, all identified variants must reach the genome-wide significance threshold (*p* < 5 × 10^−8^) and the association of identified variants needs to be confirmed in independent replication sets of cases and controls. Additionally, data from several GWAS studies can be pooled in meta-analysis leading to the discovery of variants with even smaller effect sizes. Typically, GWAS examine hundreds of thousands of SNPs in at least a thousand cases and a thousand controls, and replicate in series that are even larger [20].

### 3.2. GWAS for Bladder Cancer Risk

So far, three GWAS for UBC risk have been conducted. Firstly, a European GWAS was led by the Radboud University Nijmegen Medical Center (The Netherlands) and deCODE Genetics (Iceland), resulting in the identification of 3 UBC risk loci. The three identified loci are located in *TP63* (rs710521), *MYC* (rs9642880) [21] and *FGFR3/TACC3* (rs798766) [22]. The second GWAS was conducted by the MD Anderson Cancer Center (USA) leading to the discovery of a SNP in the *PSCA* locus (rs2294008) [23]. The US National Cancer Institute performed a third GWAS, in which three susceptibility loci were revealed: *CBX6/APOBEC3A* (rs1014971), *CCNE1* (rs8102137), and *UGT1A* (rs11892031). The association of *NAT2* variants and the *GSTM1* deletion, discovered through candidate gene studies (see above), were confirmed [24]. The association of the *SLC14A1* locus was independently reported by two groups: Rafnar *et al.* [25] and Garcia-Closas *et al.* [26]. Finally, 2 SNPs in the *TERT-CLPTM1L* region (rs2736098; rs401681), previously identified by a GWAS for basal carcinoma of the skin, were also found to be associated with the risk of UBC by targeted genotyping of selected variants [27]. A summary of all identified UBC risk loci is shown in Table 1.

## 4. Functional Studies of Risk SNPs in Bladder Cancer

Although GWAS have led to discovery of several UBC risk variants, evaluating the role of these SNPs remains challenging. Firstly, the identified variants are unlikely causative by themselves, therefore further fine-mapping, in search of stronger associated variants in the identified region, needs to be performed. Secondly, the SNPs are located mostly in the intronic and intergenic regions, and their role is still poorly understood [3]. Until now, only two of the UBC risk loci have been functionally characterized.

### 4.1. rs2294008 (PSCA)

The prostate stem cell antigen (*PSCA*) gene encodes the glycosylphosphatidylinositol (GPI)-anchored cell surface protein belonging to the *Thy-1*/*Ly-6* family. *PSCA* is expressed in a variety of normal tissues including prostate, kidney, skin, esophagus, stomach and placenta. Alterations in *PSCA* expression were described in prostate cancer, renal cell carcinoma, pancreatic cancer, ovarian cancer, non-small-cell lung carcinoma, esophageal and gastric cancer [29]. In normal urothelium, *PSCA* is expressed at low levels and the gene is highly over-expressed in the majority of NMIBC and MIBC [30]. Although *PSCA* function is unknown, other members of the *Thy-1*/*Ly-6* family, including *PSCA*, have been implicated in tumorigenesis. They are involved in cell adhesion, signal transduction, apoptosis, or T cell activation. It is hypothesized that *PSCA* may regulate self-renewal and proliferation of progenitor/stem cells [31]. Because of frequent *PSCA* over-expression in both primary and metastatic prostatic cancer, PSCA was chosen as a target for immunotherapy. It was shown that anti-PSCA antibodies are able to inhibit tumor development by reducing tumor growth and metastasis. However, the precise role of *PSCA* in this process remains to be determined [32].

**Figure 1:**
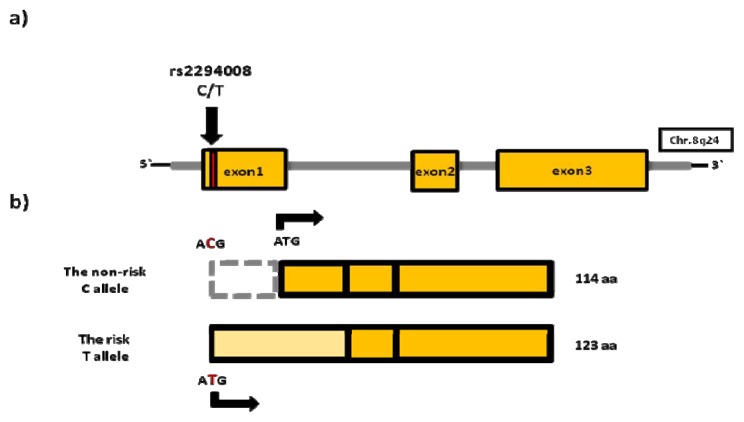
(**a**) *PSCA* locus and location of rs2294008; (**b**) Influence of rs2294008 on PSCA protein length.

The rs2294008 (C/T) SNP, located in the 8q24.3 region is, until now, the most well-studied bladder cancer susceptibility variant. This missense variant is located in exon 1 of *PSCA* and causes a change of a nucleotide in the initiation codon. As a result, a novel ATG is used for translation initiation, leading to expression of a protein 9 amino acids (aa) longer (Figure 1).

Rs2294008 has been identified previously to be associated with diffuse-type gastric cancer in the Japanese population. It was shown that the risk T allele reduced *PSCA* transcriptional activity, however no difference in the protein localization between haplotypes containing either the C or T allele was observed [33]. By contrast, a study by Tanikawa *et al.* showed that rs2294008 altered cellular localization and stability of PSCA in an allele-specific manner [34]. In bladder cancer cell lines, all haplotypes containing the risk T allele were shown to have reduced *PSCA* promoter activity, in comparison to the non-risk C allele, confirming the aforementioned results. These data suggest that the SNP in exon 1 of *PSCA* is also important for the transcription initiation of *PSCA* by, for example, affecting the binding of transcription factors (TFs) in an allele-specific manner. Unfortunately, no correlation of rs2294008 with *PSCA* mRNA expression was detected in bladder cancer cell lines [23]. To study tissue-specific gene regulatory effects, Fu *et al.* evaluated the *PSCA* mRNA expression in normal bladder and bladder cancer tissue specimens. Surprisingly, the risk T allele was found to be correlated with increased *PSCA* mRNA expression in both tissue types. Moreover, *PSCA* mRNA expression was also found to be strongly up-regulated in bladder cancer samples in comparison to adjacent normal tissue. Additionally, the authors fine-mapped the 8q24 region by imputation, and found a second variant (rs2978974) located in the alternative exon 1 of *PSCA.* This variant was found to be associated with UBC, although this still has to be confirmed in an independent replication series [35]. Rs2294008 was shown to be associated with allelic expression imbalance in normal and tumor samples, which are heterozygous for rs2294008. In normal and tumor tissue, transcripts containing the T allele were far more abundant than transcripts containing the C allele. On the protein level, cells transfected with allele-specific *PSCA* constructs showed higher PSCA cell surface expression when the risk T allele was present. Importantly, rs2294008 was also found to be strongly associated with PSCA protein expression levels in a large set of bladder tumors.

Taken together, the functional studies of *PSCA* variants indicate that *PSCA* is subjected to allele-specific regulation by rs2294008. The results suggest the possibility of a genotype-based patient selection for *PSCA* immunotherapy [36]. However, the role of *PSCA* in bladder cancer as well as the mechanism by which rs2294008 affects *PSCA* mRNA expression is unknown. Recently, the concept of the androgen-sensitive bladder cancer including *PSCA* and rs2294008 was proposed. Firstly, UBC is diagnosed 3–4 times more frequently in men, but female gender is associated with a worse prognosis [37]. Secondly, studies in mice indicated that gender-specific differences in bladder cancer susceptibility may be regulated by androgen signaling. It was shown that, when exposed to aromatic amines, male mice were more susceptible for bladder cancer than females and castrated males. None of androgen receptor (*AR*) knockout (ARKO) mice developed bladder tumors [38]. Although reports about *AR* expression in bladder cancer are conflicting [39] and rs2294008 is located outside of the androgen responsive element (ARE) mapped within the *PSCA* promoter, it can be hypothesized that other variants linked to rs2294008 can influence bladder cancer risk by lowering affinity of the AR to ARE in the *PSCA* promoter region. This event can possibly predispose cancer cells to escape from androgen regulation, leading to more aggressive and metastatic disease [40]. This hypothesis remains to be proven, but the possible influence of androgens on bladder cancer development and progression may lead to the future development of new therapies targeting hormonal signaling [41].

### 4.2. rs17863783 (UGT1A)

The UDP-glucuronosyltransferase (*UGT*) 1A locus is located on chromosome 2q37. This complex gene is subjected to extensive alternative splicing, leading to the presence of at least nine functional isoforms. Each isoform has a unique alternative exon 1, whilst exons 2–5 are shared among all isoforms [42]. The *UGT1A* isoforms are expressed in the majority of tissues, including the liver, kidney, gastrointestinal tract, and the urinary bladder [43]. The *UGT1A* family is involved in the detoxification of many endogenous (steroids, bilirubin) and xenobiotic (drugs, carcinogens) substrates, mainly in the liver but also in extrahepatic tissues [44]. They are involved in inactivation of aromatic amines through glucoronidation, which increases the solubility and excretability of these compounds. However, the *N*-glucoronidation facilitated by UGT1A is reversible in the acidic urine, leading to the accumulation of aromatic amines in the urothelium and formation of DNA mutagenic adducts which contribute to carcinogenesis [45]. Moreover, exposure to aromatic amines (e.g., by smoking and certain occupations) is a known risk factor for developing bladder cancer [46]. Genetic variants in the aromatic amines detoxification enzymes have been identified to be associated with bladder cancer risk, including the *GSTM1* deletion (null genotype) and the *NAT2* slow acetylator genotypes [28]. Additionally, genetic variants in *UGT1A* have been implicated in the modulation of toxicity of the chemotherapeutic agent irinotecan, influencing the clinical outcome of treatment [47].

**Figure 2:**
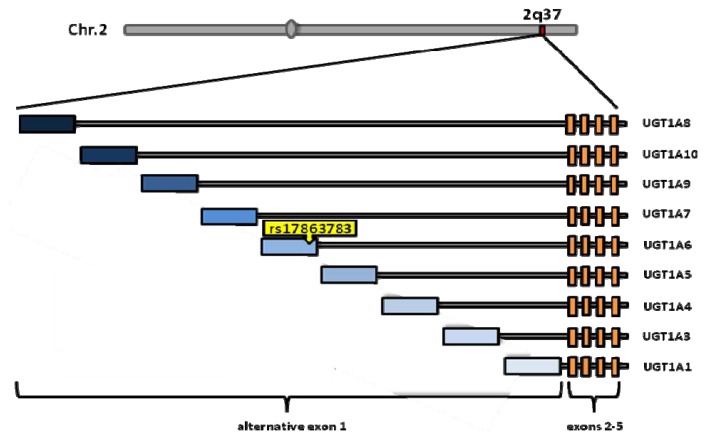
*UGT1A* locus and location of the functional UBC variant.

One of bladder cancer susceptibility loci maps to *UGT1A*. Rs11892031 (A/C), located in intron 1 of *UGT1A8* and *UGT1A10*, has been discovered by Rothman *et al.* [24]. In follow-up studies, fine-mapping of the region was performed by re-sequencing, leading to the identification of a causative genetic variant rs17863783 (G/T), which explained the association in this region found by GWAS. This synonymous variant is located in the long isoforms of *UGT1A6* (*UGT1A6.1*) (Figure 2).

Although this variant does not change the amino acid sequence, Tang *et al.* hypothesized that it can modify the *UGT1A6.1* expression level by influencing the exonic splicing enhancer (ESE) in an allele-specific manner. In an *in vitro* experiment, the presence of the protective T allele caused a significant increase in *UGT1A6.1* expression, compared to the G allele. Due to the fact that no carriers of the uncommon T allele (MAF = 0.025) were detected in bladder samples, rs17863783 was correlated with *UGT1A6.1* expression in normal liver samples. The protective T allele was found to be correlated with increased expression of *UGT1A6.1*. The increased expression of *UGT1A6* in bladder tissue can lead to enhanced removal of carcinogens dissociated from glucoronids from bladder tissue into the urine, thereby reducing bladder cancer risk [48].

Several studies have shown the importance of enzymes involved in the detoxification of aromatic amines and other carcinogens in bladder cancer susceptibility [49]. Tang *et al.* have provided compelling evidence that a variant in the *UGT1A6.1* gene is functional and affects RNA splicing and gene expression in an allele-specific manner. This effect of a genetic variant on *UGT1A6.1* gene expression may lead to enhanced detoxification of the bladder tissue and therefore to a reduced bladder cancer risk.

### 4.3. Functional Studies of Risk SNPs in UBC Susceptibility Regions

#### 4.3.1. *MYC* Locus on 8q24

The v-myc myelocytomatosis viral oncogene homolog (*MYC*) gene is one of the most well-studied proto-oncogenes. This transcription factor regulates hundreds to thousands of genes involved in a wide variety of biological processes, both in normal and malignant cells [50]. *MYC* is known to influence proliferation, apoptosis, differentiation, self-renewal/senescence and angiogenesis [51]. It is also one of the most frequently up-regulated genes in cancer, suggesting its importance in tumorigenesis [52]. Increased *MYC* expression in tumors was found to be caused by different mechanisms, including *MYC* gene amplification, chromosomal translocations, changes in the *MYC* regulatory network, and mutations targeting the upstream signaling pathways or MYC protein stability [53].

The *MYC* gene is located in the 8q24 region, in which several susceptibility loci were identified by GWAS for many epithelial cancers including colorectal cancer [54], prostate cancer [55], breast cancer [56] and bladder cancer. The bladder cancer susceptibility variant rs9642880 (G/T) is located 30 kb upstream of the *MYC* gene and is in weak LD with the other risk variants on the 8q24. No correlation of rs9642880 with *MYC* expression was detected in whole blood or adipose tissue. It is, however, possible that the *MYC* regulatory mechanism, which may be modified by rs9642880, is tissue-specific and therefore detectable only in urothelium [21]. The association of rs9642880 was replicated in a Chinese population and it was shown that the risk T allele was correlated with increased *MYC* mRNA expression in normal bladder tissue and protein expression in tumors [57]. These data suggest that this variant or a variant in high LD is indeed functional. Until now, no functional studies of rs9642880 have been published. On the other hand, several functional studies have been performed on SNPs that are scattered over a 500 kb region upstream of the *MYC* gene, and that are associated with several cancer types. No transcriptional (*i.e.*, promoter) activity of the 8q24 risk-alleles was detected in colorectal, prostate or breast cancer. However, it was shown that the risk loci, located far upstream of *MYC*, were shown to physically interact with the *MYC* promoter in a tissue-specific manner, possibly influencing *MYC* expression levels [58]. Furthermore, the risk variants lie within regions containing chromatin marks for regulatory elements and were shown to act as enhancers [59,60]. The most extensively studied genetic variant in the 8q24 gene desert upstream of *MYC* gene is the colorectal and prostate cancer risk SNP rs6983267. It was shown that rs6983267 maps to a highly conserved TCF4 transcription factor binding site within a functional enhancer region (MYC-335). TCF4 is known to play a role in Wnt signaling (frequently deregulated in colorectal cancer) and *MYC* is one of the target genes of the Wnt signaling pathway [61]. Indeed, TCF4 binding at rs6983267 was found to be allele-specific, with increased affinity to the risk G allele. The rs6983267 region was found to be able to interact with the *MYC* promoter via chromatin looping, but no difference in efficiency of chromatin looping between alleles was observed. Conversely, rs69833267 was found to regulate enhancer activity in an allele-specific manner *in vitro* [62,63] and *in vivo* in transgenic mice [64]. Despite the reported enhancer activity of the rs6983267 locus, no correlation with *MYC* expression has been found so far, neither in colorectal nor in prostate cancer tissue samples [65]. Interestingly, Sur *et al.* showed that the presence of the MYC-355 enhancer, containing rs6983267, is not crucial for normal intestinal development and function. Surprisingly, MYC-355 only seems to be involved in tumorigenesis, as lack of the enhancer region reduced the formation of polyps in an Apc^min^ mouse strain that spontaneously develops colorectal tumors [66].

Studies in the other cancer types show that the risk variants at the 8q24 are able to affect *MYC* promoter activity in an allele-specific manner, by influencing transcription factors binding affinity. It is therefore reasonable to hypothesize that rs9642880 lies within such a regulatory region and that altered TFs binding may modify *MYC* expression in a similar way. Given that frequent *MYC* over-expression or amplification has been described in bladder cancer [67], *MYC* seems to play an important role in bladder cancer development. Many therapies targeting *MYC* expression, function, or interaction with other proteins, are currently being developed [68]. Thus, future studies elucidating the role of *MYC* and rs9642880 in bladder cancer may have an impact on the selection of patients who may benefit from *MYC*-targeted therapies.

#### 4.3.2. *TERT/CLPTM1L* Locus on 5p15

Multiple studies linked numerous variants at the 5p15 region to cancer risk, including breast cancer, pancreatic cancer, and glioma [69]. Furthermore, two bladder cancer risk variants in the 5p15 were discovered including rs2736098, a synonymous variant located in the exon of the *TERT* gene and rs401681 in intron 4 of *CLPTM1L*. Additionally, both variants were found to be associated with increased risk of basal cell carcinoma, lung cancer and cervical cancer, which are known to be strongly connected to environmental exposures. Interestingly, rs401681 was identified as a protective variant for melanoma [27].

Telomerase is a ribonucleoprotein, which consists of the integral telomerase RNA (*TER*) and telomerase reverse transcriptase (*TERT*). Telomerase prevents loss of the chromosomal ends, which occurs during DNA replication. This, in turn, ensures genome stability and integrity. In normal cells telomerase is expressed at low levels and it is tightly controlled through transcriptional and epigenetic regulation. The expression of telomerase decreases with subsequent cell divisions, leading to an increase in telomeres shortening. Telomere shortening eventually leads to senescence and apoptosis [70,71]. The balance between telomere shortening and maintenance is known to influence cancer risk [72]. Remarkably, telomerase (*TERT*) expression is deregulated in approximately 85% of cancers, through *TERT* amplification or 5p chromosomal gains. The up-regulation of *TERT* expression leads to maintenance of telomeres length, resulting in unlimited proliferation and immortalization of cancer cells, one of the hallmarks of carcinogenesis [73]. A second gene in the 5p15 locus, upstream of *TERT* is, the less-studied cleft lip and palate transmembrane protein 1-like protein (*CLPTM1L*). *CLPTM1L* was shown to influence the sensitivity of ovarian cancer cells to cisplatin-induced apoptosis [74]. It was also found to be over-expressed in doxorubicin-resistant breast cancer, although the mechanism by which *CLPTM1L* affects drug resistance is unknown [75].

Based on the importance of *TERT* in carcinogenesis, several studies aimed to evaluate the influence of discovered variants. In prostate cancer, fine-mapping of the original GWAS signal was performed, leading to the identification of four distinct *TERT* regions independently associated with prostate cancer risk. Interestingly, the identified regions were found to be located within the active regulatory elements predicted by The Encyclopedia of DNA Elements (ENCODE). Additionally, SNPs in one of the identified risk regions influenced *TERT* mRNA expression in benign prostate tissue [76]. In the previously mentioned study by Rafnar *et al.* the risk alleles of the two identified SNPs were found to be correlated with a shorter length of telomeres in blood of older, but not younger, women. It was therefore hypothesized that risk variants may affect telomere length gradually over time, leading to increased genomic instability of proliferating cells [27]. The telomere length is known to modify bladder cancer risk and is affected by cigarette smoking [77,78]. By contrast, studies in lung cancer showed that risk variants in *TERT* are associated with longer telomeres, possibly promoting tumorigenesis by increasing the number of cell divisions, leading to acquisition of more somatic changes in DNA [79]. Recent studies in breast and ovarian cancer indicated, however, that regulation of *TERT* expression and the role of the identified risk variants could be much more complex. The risk SNPs in the *TERT* region were shown to differentially influence *TERT* promoter activity and affect telomere lengths. For example, it was shown that some variants did not affect telomere length, but silenced *TERT* promoter activity and led to expression of an alternatively spliced, truncated *TERT* product [80]. The impact of genetic variation on *CLPTM1L* expression is less studied. Recently, however, it was shown that the risk allele of the intronic rs402710 was associated with increased levels of DNA adduct accumulation in lung tissue. This suggests that variants identified in *CLPTM1L* are involved in metabolism of carcinogens present in, for example, tobacco smoke [81]. A study by James *et al.* showed that *CLPTM1L* is frequently over-expressed in lung cancer. In addition, *CLPTM1L* was shown to play an anti-apoptotic role by protecting cancer cells from genotoxic-stress induced DNA damage [82].

In conclusion, the 5p15 locus was frequently identified to be associated with increased risk for different cancer types, suggesting an important role in cancer etiology. Firstly, it can be hypothesised that rs2736098 and/or other variants in LD with rs2736098 can influence *TERT* transcription levels or telomerase activity, leading to increased UBC susceptibility. As new therapies targeting *TERT* signaling are being developed [83], elucidating *TERT* regulation in UBC is of particular interest. Secondly, considering that smoking is a strong risk factor for developing lung and bladder cancer, it is possible that the risk variants identified at the 5p15 locus affect the *CLPTM1L* gene, thereby influencing the metabolism of carcinogens, and hence modify cancer risk. So far, functional studies on the *TERT* and *CLPTM1L* variants in other cancer types have given conflicting data. Therefore, detailed studies are needed to evaluate the role of the 5p15 risk SNPs in UBC development and to unravel their possible interaction with environmental risk factors.

## 5. Future of Functional Studies

### 5.1. Possible Role of Variants in Non-Coding Regions

Until now, the majority of UBC risk variants have not been functionally characterized. However, an increasing amount of data is published about the role of the risk variants in modification of cancer susceptibility. Variations in the human genome can influence cellular processes on different levels. Exonic genetic variants may affect protein folding, cellular localization, stability, or expression, and may disrupt splicing. Intronic and intergenic variants may influence, amongst others, gene expression regulation by alterations in transcription factors binding sites, microRNAs sequence or target sites, non-coding RNAs or RNA splicing.

Frequent mutations in the protein coding regions, involved in tumor development, have been described in many cancer types [84], including bladder cancer [85]. The majority of GWAS signals map to the still poorly understood non-coding regions of the genome. Initiatives like ENCODE proved that large parts of the genome, including non-coding regions, are transcribed and probably functional [86]. Over 75% of the GWAS risk variants were shown to map to regulatory elements or to be in strong LD with SNPs in active regions [87]. Genetic variants regulate gene expression in a *cis*- (local) and *trans*- (distal) manner, resulting in heritable differences in gene expression levels between individuals, which may be found through expression quantitative trait loci (eQTLs) analysis [88]. Several studies in different cancer types confirmed that, indeed, the intergenic risk variants map to novel, tissue-specific regulatory elements. Furthermore, these SNPs were shown to alter the affinity of transcription factors binding, leading to an allelic imbalance in the target gene expression [89–91]. Secondly, it is known that long non-coding RNAs (lncRNAs) regulate a large variety of biological processes and play important roles in cancer development and, as such, these lncRNAs can act as oncogenes or tumor-suppressors [92]. Some risk variants identified by GWAS were found to influence the expression of tissue-specific lncRNAs in an allele-specific way [93]. For example, Jendrzejewski *et al.* showed that the non-coding papillary thyroid carcinoma risk variant maps to the novel, thyroid-specific, long intergenic non-coding RNA, *PTCSC3*. The expression of this tumor-suppressor RNA was found to be strongly down-regulated in cancer in an allele-specific manner, leading to an increase in cell growth [94]. Thirdly, alternative splicing is known to be frequently deregulated in cancer and will lead to exon skipping or aberrantly excised intronic sequences [95]. Consequently, altered protein expression may influence proliferation, survival and metastatic potential of cancer cells [96]. Several SNPs in the exonic and intronic regions were shown to influence the patterns of splicing by altering the splicing regulatory sequences, including the exonic and intronic splicing enhancers or silencers. In prostate cancer the intronic risk SNP was shown to lie within a splicing enhancer region of the *KLF6* tumor-suppressor gene. This variant was found to increase the transcription of alternatively spliced *KLF6* isoforms, which act in a dominant-negative way on wild-type *KLF6*, resulting in increased transcriptional activation of growth stimulating genes [97]. Fourthly, microRNAs (miRNAs) are post-transcriptional regulators of gene expression and are known to be involved in regulation of a wide range of genes and biological processes [98]. Kim *et al.* showed that SNPs located in miR-1206 at the 8q24 and miR-612 at the 11q13.3 regions influence the biogenesis of the corresponding mature miRNAs in an allele-specific manner [99]. Furthermore, it was shown that in esophageal squamous cell carcinoma, a risk SNP in the 3′UTR of the *RAP1A* gene, affects the binding site of miRNA-196a. This led to *RAP1A* over-expression resulting in more aggressive tumor behavior [100].

Taken together, the GWAS results suggest that cancer susceptibility is in general modified by the alterations or variations in gene expression regulatory mechanisms, rather than by amino acid sequence changes. Several diseases have already been linked to mutations in non-coding regions including Hirschsprung disease [101], colorectal cancer [102], or melanoma [103,104], pointing towards a crucial role of *cis*-regulatory regions in health and disease [105]. Considering the rapid development of the techniques suitable for analysis of the non-coding regions, as well as the more accurate and more comprehensive annotation of tissue-specific regulatory elements, functional studies of the intronic and intergenic variants will become more straightforward in the future [106].

### 5.2. Rapid Development of New Technologies

Despite the success of GWAS in the identification of novel risk loci, only a small part of an individual’s cancer susceptibility can be explained by genetic variants. The majority of studies so far focused on common variants following the “common disease–common variant” hypothesis. With improvements in technologies the “missing heritability” problem is expected to be (at least partly) solved by the discovery of multiple low-effect size, rare and/or structural variants associated with a particular disease [107].

Initiatives like The International 1000 Genomes Project will improve the characterization of human genome variation by sequencing individuals from different populations, leading to the annotation not only common of variants (MAF > 5%), but also low frequency (MAF < 1%) and rare variants (MAF < 0.5%) [108,109]. In the past years, genotyping chips allowed for the detection of common variants. Recently, new chips have been designed that allow the detection of less common variants. However, next generation sequencing, including whole-genome and whole-exome sequencing, are emerging as new, powerful tools in the detection of rare variants [110]. It is possible that multiple low penetrance variants associated with disease remain to be discovered. Larger GWAS initiatives and meta-analyses of the existing data are needed in order to discover the common variants with even smaller effects contributing to overall disease susceptibility [111]. Despite improvements in technologies that detect copy number variants (CNVs) through SNP arrays, calling of CNVs pose a particular challenge. With further developments of methodology and annotation of CNVs our understanding of the full spectrum of this variation will become more complete [112].

### 5.3. The Impact of Context

Until now, the bladder cancer risk loci confer only a small increased risk, suggesting that pathways involved in cancer development can be much more complex than expected and that the risk variants need to be studied in particular cancer subtypes or environmental context.

The number of identified risk loci differs significantly between cancer types. This highlights the polygenic nature of cancer and the differences in etiology between different tumors. Alternatively, some of the tumor types can be more heterogeneous than others, which complicates the discovery of more general variants [113]. Secondly, GWAS risk signals show a remarkable subtype specificity. In breast cancer, several risk variants were shown to be associated only with the more aggressive ER-negative tumors but not with ER-positive cancer, confirming observed differences in clinical behavior of both tumor types [114]. Interestingly, several bladder cancer risk variants including *MYC* [21] and *FGFR3*/*TACC3* [22] show stronger association with low risk tumors, confirming the heterogeneous nature of bladder cancer [115]. In this regard, identification of pathways affected by subtype-specific variants and involved in less or more aggressive disease development will be important for cancer prevention. The collection of larger sample sizes corresponding to disease subtypes can result in the identification of more loci which are subtype-specific [116]. Finally, interactions between identified SNPs and environmental and lifestyle factors can modify individual cancer susceptibility, but this has not yet been extensively studied [117]. Garcia-Closas *et al.* showed that 6 of 12 UBC susceptibility variants showed significant additive gene–environment interactions, most notably true for *NAT2* and *UGT1A6*. They also demonstrated how GWAS data can be used for predicting the absolute risk of bladder cancer, taking into account smoking [118]. It would be of particular interest to study the additive effects of the identified UBC susceptibility variants in other environmental and lifestyle contexts, although it requires large sample sizes and above all valid phenotypic and exposure information [119].

## 6. Conclusions

The GWAS approach has been successful in the identification of UBC risk genetic variants. Until now, only two of the identified loci (*PSCA* and *UGT1A*) have been functionally characterized. These studies, however, confirmed that variants identified by GWAS have functional consequences and influence gene expression in an allele-specific manner. Furthermore, the GWAS approach pointed towards new genes involved in bladder cancer development, possibly leading to the development of new therapies in the future. In this regard, the heritable differences in gene expression related to risk SNPs have potential to be used in the selection of patients who will benefit from a particular treatment. As our understanding of the non-coding regions is still far from complete, the role of other bladder cancer risk variants remains unknown. An increasing amount of data is being published about the influence of intergenic and intronic SNPs in modification of various biological processes. It can, therefore, be hypothesized that UBC risk variants affect gene expression by alterations in urothelium-specific regulatory networks through one of the aforementioned mechanisms. A large part of UBC susceptibility remains to be uncovered. Bladder cancer occurs frequently and is associated with high economic costs, due to extensive follow-up [120]. Screening programs for bladder cancer are currently not thought to reduce disease specific outcomes. Tailoring of such programs according to genetic risks and patterns of exposure to carcinogens may enable the identification or selection of people at risk [121]. Until now, individual genetic risk assessment using the risk variants has not been clinically relevant, however, it cannot be excluded that, with increasing numbers of identified variants, genetic counseling of high risk groups, based on GWAS results, will become possible [122]. In conclusion, GWAS results are very promising in bringing us closer to the era of personalized medicine. However, they still pose a particular challenge in the interpretation of their functional significance.

## Figures and Tables

**Table 1:** Urinary bladder cancer risk variants.

Chrom.	Gene	SNP (identified allele)	Location	MAF	OR (95% CI)	Study type	Ref.
8q24	*MYC*	rs9642880 [T]	Intergenic	0.45	1.22 (1.15–1.29)	GWAS	[21]
3q28	*TP63*	rs710521 [A]	Intergenic	0.73	1.19 (1.12–1.27)	GWAS	[21]
5p15	*CLPTM1L*	rs401681[C]	Intronic	0.53	1.12 (1.06–1.18)	Follow-up GWAS	[27]
5p15	*TERT*	rs2736098 [A]	Exonic	0.25	1.16 (1.08–1.23)	Follow-up GWAS	[27]
8q24	*PSCA*	rs2294008 [T]	Exonic	0.46	1.15 (1.10–1.20)	GWAS	[23]
4p16	*FGFR3/TACC3*	rs798766 [T]	Intronic	0.19	1.24 (1.17–1.32)	GWAS	[22]
22q13	*CBX6/APOBEC3A*	rs1014971 [C]	Intergenic	0.38	0.88 (0.85–0.91)	GWAS	[24]
19q12	*CCNE1*	rs8102137 [C]	Intergenic	0.33	1.13 (1.09–1.17)	GWAS	[24]
2q37	*UGT1A*	rs11892031 [C]	Intronic	0.08	0.84 (0.79–0.89)	GWAS	[24]
18q12	*SLC14A1*	rs17674580 [T]	Intronic	0.33	1.17 (1.11–1.22)	GWAS	[25]
18q12	*SLC14A1*	rs7238033 [T]	Intronic	0.43	1.20 (1.13–1.28)	Meta-analysis	[26]
1p13	*GSTM1*	Null *	–	0.51	1.5 (1.3–1.6)	Candidate gene, meta-analysis	[28]
8p22	*NAT2*	Slow acetylator *	–	0.56	1.4 (1.2–1.6)	Candidate gene, meta-analysis	[28]

* NAT2 slow acetylator and GSTM1 null refer to the risk genotypes not the risk SNPs; MAF: Minor allele frequency; OR: Odds ratio; CI: Confidence intervals.
